# Incidence of Type 2 Diabetes in Pre-Diabetic Japanese Individuals Categorized by HbA_1c_ Levels: A Historical Cohort Study

**DOI:** 10.1371/journal.pone.0122698

**Published:** 2015-04-08

**Authors:** Tetsuya Kawahara, Ryuichiro Imawatari, Chie Kawahara, Tetsuya Inazu, Gen Suzuki

**Affiliations:** 1 Kokura Medical Association Health Testing Center, Kitakyushu, Fukuoka, Japan; 2 Kitakyushu Medical Association Committee on Health Promotion, Kitakyushu, Fukuoka, Japan; 3 Kokura Medical Association, Kitakyushu, Fukuoka, Japan; 4 First Department of Internal Medicine, School of Medicine, University of Occupational and Environmental Health, Kitakyushu, Fukuoka, Japan; 5 Department of Pharmacy, Ritsumeikan University, Kusatsu, Shiga, Japan; 6 Department of Internal Medicine, International University of Health and Welfare Clinic, Otawara, Tochigi, Japan; National University of Singapore, SINGAPORE

## Abstract

**Objective:**

Reported incidence of type 2 diabetes estimated at the pre-diabetic stage differs widely (2.3–18.1% per year). Because clinicians need to know the risk of incident diabetes after a diagnosis of pre-diabetes, our objective was to estimate precise incidence of diabetes using baseline HbA_1c_ levels.

**Methods:**

A historical cohort study using electronic medical record data obtained between January 2008 and December 2013. A total of 52,781 individuals with HbA_1c_ < 6.5% were assigned to one of six groups categorized by baseline HbA_1c_ level: ≤ 5.5% (n=34,616), 5.6–5.7% (n=9,388), 5.8–5.9% (n=4,664), 6.0–6.1% (n= 2,338), 6.2–6.3% (n=1,257), and 6.4% (n=518). Participants were tracked until a subsequent diagnosis of diabetes or end of follow-up during a period of 5 years.

**Results:**

During the follow-up period (mean 3.7 years), 4,369 participants developed diabetes. The incidence of diabetes in the first year was 0.7, 1.5, 2.9, 9.2, 30.4, and 44.0% in the six HbA_1c_ groups, respectively. At five years the incidence was 3.6, 8.9, 13.8, 27.5, 51.6, and 67.8%, respectively (p < 0.0001 comparing the HbA_1c_ ≤5.5% group to the other groups). After adjustment for confounding factors, the hazard ratios compared with the HbA_1c_ ≤5.5% group were significantly elevated: 2.3 (95%CI 2.0–2.5), 3.4 (95%CI 2.9–3.7), 8.8 (95%CI 8.0–10.1), 26.3 (95%CI 23.3–30.1), and 48.7 (95%CI 40.8–58.1) in the five HbA_1c_ groups (p < 0.0001).

**Conclusion:**

By fractionating baseline HbA_1c_ levels into narrower HbA_1c_ range groups, accuracy of estimating the incidence of type 2 diabetes in subsequent years was increased. The risk of developing diabetes increased with increasing HbA_1c_ levels, especially with the HbA_1c_ level ≥ 6.2% in the first follow-up year.

## Introduction

Diabetes mellitus is a major burden to healthcare systems and societies worldwide [1–6]. The increasing economic burden of diabetes results in large part from the increase in the number of people with diagnosed diabetes. Five hundred and ninety two million people worldwide will have type 2 diabetes by the year 2030 [7]. From the perspectives of public health, medical care, and economics, it is preferable to detect individuals at high risk for diabetes (*i*.*e*., pre-diabetes: impaired fasting glucose and/or impaired glucose tolerance) early, and initiate interventions such as lifestyle modification.

The incidence of type 2 diabetes among pre-diabetic individuals has been reported and incident rates differ greatly from 2.3% to 18.1% per year [8–14]. Although there are many previous studies that estimated the incidence of diabetes based on baseline HbA_1c_ levels, those levels were roughly categorized by increments of 0.5% or more [8,10–12,14], resulting in widely differing and less accurate estimated incidences. If properly educated, pre-diabetic individuals may prevent the occurrence of type 2 diabetes by implementing lifestyle changes. Therefore, it is important for both clinicians and pre-diabetic individuals to be empowered by knowing the precise risk of diabetic incidence based on measured HbA_1c_. Hence we conducted a historical cohort study to investigate the incident risk of diabetes during 5 years of follow-up by dividing baseline HbA_1c_ levels into 6 narrower intervals— ≤ 5.5%, 5.6–5.7%, 5.8–5.9%, 6.0–6.1%, 6.2–6.3%, and 6.4%—using electronic medical record data obtained from annual medical examinations.

Somewhat unique to Japan, most adults undergo an annual medical examination either in their work place or in the community. During the examination, fasting plasma glucose and/or HbA_1c_ levels are measured to evaluate the status of glucose metabolism. Using those data, our study could provide more precise information about the magnitude of diabetes risk in pre-diabetic subjects.

## Methods

### Study design

This historical cohort study was community based, including 60,975 participants at one healthcare center and 27 clinics. All participants had an HbA_1c_ test during at least one annual medical examination in the baseline period, January 1, 2008 to December 31, 2008. They were interviewed by trained nurses at the time of each annual examination using standard questionnaires to gather information about demographic characteristics, medical history, and health-related habits. Height, weight, and blood pressure measurements as well as laboratory tests were conducted at the same time.

Every blood specimen was assayed for HbA_1c_ levels in the same laboratory (Kitakyushu Central Laboratory Testing Center, Kitakyushu, Japan). High-performance liquid chromatography using an ADAMS A1c automatic glycohemoglobin analyzer (ARKRAY, Kyoto, Japan) was performed to analyze samples from January 1, 2008 to April 11, 2010. Latex agglutination immunoassay using a DM-JACK Ex. analyzer (KYOWA MEDEX, Tokyo, Japan) was performed from April 12, 2010 to December 31, 2013. Therefore, all the HbA_1c_ measurements at baseline were performed by High-performance liquid chromatography, while measurements during the follow-up period were done by two methods. The accuracy of the equipment used to measure HbA_1c_ was also validated. At an HbA_1c_ of 6.4%, the standard error (SE) was 0.015, and the 95% confidence interval (CI) was 6.371–6.429%. The SEs were from 0.0131 to 0.0162 for the other HbA_1c_ levels. The 95% CIs for HbA_1c_ levels were within ± 0.0318%. Therefore, the probability that participants were assigned to the wrong baseline HbA_1c_ group was < 0.05. The study endpoint was whether a diagnosis of diabetes occurred during the 5-year follow-up period.

The values for HbA_1c_ (%) in Japan were estimated using the Japan Diabetes Society (JDS) definition until April 1, 2012. We used the following formula to convert from the JDS value to the National Glycohemoglobin Standardization Program (NGSP) equivalent: NGSP (%) = JDS (%) + 0.4 [15] and evaluated all HbA_1c_ data using NGSP values.

### Ethics statement

This study was approved by the Review Boards of the Kokura Medical Association Health Testing Center and the Kitakyushu Medical Association Committee on Health Promotion. The study was conducted according to the principles expressed in the Declaration of Helsinki. Participants did not provide verbal or written informed consent at the time of examination, but were allowed to refuse participation. This procedure conforms to the Japanese Ethical Guidelines for Epidemiological Research; informed consent is not strictly required for observational studies using existing data, but researchers should disclose information on the objective and conduct of the study and provide prospective subjects an opportunity to refuse inclusion of their data in the research [16]. Participant’s records/information was anonymized and de-identified prior to analysis.

### Subjects

Participants comprised mainly Japanese residents and workers in Kitakyushu city who underwent annual examinations for health screening. Inclusion criteria were that they were apparently healthy and had attended at least one out-patient visit at any time during the follow-up period *(i*.*e*., between the date of baseline HbA_1c_ testing and December 31, 2013) or before a diagnosis of diabetes. Participants' records were evaluated to assure that they had at least one outpatient visit, no HbA_1c_ ≥ 6.5%, no diagnosis of diabetes, and no medication for diabetes.

Using these inclusion criteria, 2,705 subjects were excluded from 60,975 potential participants for having HbA_1c_ ≥ 6.5% or having been diagnosed with diabetes, and 5,489 subjects were excluded who failed to revisit during the follow-up period (S1 Table). Subjects who had missing values for participants’ records or who were lost to follow-up after one or more revisit(s) during the follow-up period were eligible and included in the analysis (S2 Table). Thus, a total of 52,781 subjects had usable data for this study. During the follow-up period, diabetes onset was identified using an accepted method to ascertain diabetic status [17,18], *i*.*e*., HbA_1c_ ≥ 6.5%, fasting plasma glucose ≥126 mg/dl, casual plasma glucose ≥200 mg/dl, or initiation of any medication for diabetes treatment.

### Statistical analysis

Participants were divided by baseline HbA_1c_ level into 6 groups: (1) ≤ 5.5%, (2) 5.6–5.7%, (3) 5.8–5.9%, (4) 6.0–6.1%, (5) 6.2–6.3%, and (6) 6.4%. The lowest group (HbA_1c_ ≤ 5.5%) was the reference group. The other groups were compared with this reference group in the risk calculation. Descriptive statistics were used to describe the study population at baseline. The median and range were calculated for continuous variables. Frequencies and proportions were calculated for categorical variables. The baseline characteristics of subjects who developed diabetes in the follow-up period were compared with those who did not develop diabetes. The nonparametric Wilcoxon rank-sum test was used to compare medians for continuous variables. The Chi-square test was used to compare proportions for categorical variables. The Kaplan—Meier procedure was used to estimate the diabetes-free probability, and the log-rank statistic was used to test for overall differences in cumulative incidence of diabetes among the baseline HbA_1c_ levels. A Cox proportional hazards model, adjusted for age, sex, body mass index (BMI), hypertension, family history of diabetes, exercise habit, smoking habit, and elevated alcohol consumption was used to compare the risk of developing diabetes for each baseline HbA_1c_ level (using HbA_1c_ ≤ 5.5% as the reference group). Unadjusted and multivariate adjusted hazard ratios were calculated. Cox proportional hazards models were also stratified by age, sex, BMI, hypertension, or smoking habit, respectively. The Cox model was also used to test the multiplicative effect of other risk factors on HbA_1c_: age, sex, BMI, hypertension, and smoking habit. All tests were two-tailed. The Bonferroni correction was used to correct significance levels for multiple comparisons: because there were six groups and 15 comparisons, *P* values < 0.0033 (= 0.05/15) were considered to indicate statistical significance. Statistical analysis was performed using GraphPad Prism ver.5 (GraphPad software, La Jolla, CA, USA) for the Kaplan-Meier analysis and SPSS ver.15 (SPSS Inc., Chicago, IL, USA) for other analyses.

## Results

The median age was 67 years (range: 20–89 years), and 41.6% were males. Mean follow-up period was 3.7 years. During follow-up, 4,369 (8.3%) participants developed diabetes. Higher incidence of diabetes was significantly associated with male sex, BMI, blood pressure, hypertension, family history of diabetes, absence of exercise habit, smoking habit, and heavy drinking habit (p<0.0001) (Table 1).

**Table 1:** Baseline characteristics of the study population .

	All subjects	Diabetes	Non-diabetes	P value	Odds ratio (95% CI) ^¶^
n	52781	4369 (8.3%)	48412 (91.7%)		
Age (years)	67 (20–89)	67 (37–74)	67 (20–89)	0.6712	
Sex					
Female	30824 (58.4)	1874 (42.9)	28950 (59.8)	-	1.00
Male	21957 (41.6)	2495 (57.1)	19462 (40.2)	<0.0001	1.97 (1.85–2.11)
Body mass index (kg/m^2^)	23.0 (9.0–41.0)	24.0 (11.0–41.0)	23.0 (9.0–35.0)	<0.0001	1.18 (1.09–1.27)
Blood pressure (mmHg)					
Systolic	133 (82–231)	139 (88–231)	132 (82–225)	<0.0001	
Diastolic	75 (50–118)	77 (50–114)	74 (50–118)	<0.0001	
Hypertension *					
No	28100 (53.2)	1909 (43.7)	26191 (54.1)	-	1.00
Yes	24681 (46.8)	2460 (56.3)	22221 (45.9)	<0.0001	1.49 (1.39–1.58)
Family history of diabetes ^†^					
No	31285 (59.3)	1560 (35.7)	29725 (61.4)	-	1.00
Yes	21496 (40.7)	2809 (64.3)	18687 (38.6)	<0.0001	2.84 (2.67–3.04)
Exercise habit ^‡^					
No	35748 (67.7)	3264 (74.7)	34484 (67.1)	-	1.00
Yes	17033 (32.3)	1105 (25.3)	15928 (32.9)	<0.0001	0.69 (0.63–0.74)
Smoking habit ^§^					
No	42523 (80.6)	3019 (69.1)	39504 (81.6)	-	1.00
Yes	10258 (19.4)	1350 (30.9)	8908 (18.4)	<0.0001	1.96 (1.83–2.10)
Drinking habit ^||^					
No	42314 (80.2)	3342 (76.5)	38972 (80.5)	-	1.00
Yes (moderate)	2670 (5.0)	201 (4.6)	2469 (5.1)	0.446	0.95 (0.82–1.09)
Yes (heavy)	7797 (14.8)	826 (18.9)	6971 (14.4)	<0.0001	1.37 (1.29–1.53)

Values are expressed as medians (range or percentage).

* Hypertension: Yes = systolic blood pressure ≥140 mmHg, diastolic blood pressure ≥90 mmHg, or treated with anti-hypertension drugs.

^†^ Family history of diabetes: Yes = third-degree relatives with diabetes.

^‡^ Exercise habit: Yes = exercised for ≥30 min each time and two or more times per week.

^§^ Smoking habit: No = never smoked or quit smoking > 6 months ago.

^||^ Drinking habit (alcohol consumption habit): Yes (moderate) = consumed alcohol ≥ 3 per week and ≥ 180 mL sake (same as 110 mL distilled spirits (shochu), 60 mL whisky, 200 mL wine, or 500 mL beer) at a time. Yes (heavy) = consumed alcohol ≥ 3 times per week and ≥ 180 mL sake (same as 110 mL distilled spirits (shochu), 60 mL whisky, 200 mL wine, or 500 mL beer) at a time.

^¶^ Odds ratios were adjusted by age, sex, and body mass index.

95% CI: 95% confidence interval

**Fig 1:**
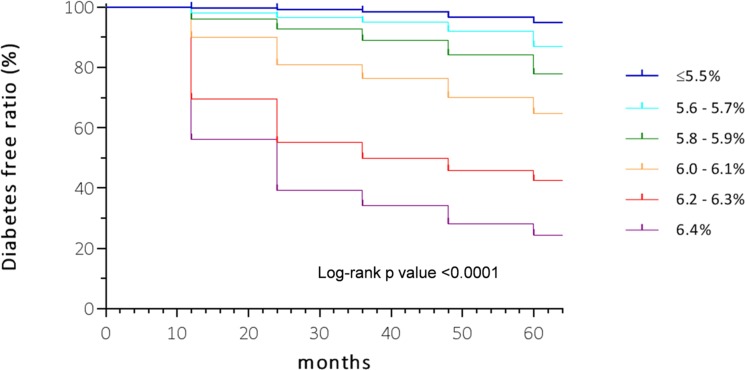
Kaplan—Meier survival curves for incident diabetes during the 5-year study period, differentiated by baseline HbA_1c_ levels. Each curve had a significantly higher risk compared with the reference group, the curve for HbA_1c_ ≤ 5.5% (log-rank P value <0.0001).

Fig 1 presents Kaplan—Meier curves of diabetes-free probability based on the baseline HbA_1c_ categories. Compared with the reference group (HbA_1c_ ≤ 5.5%), the other groups had significantly lower diabetes-free probability (log-rank *P* values < 0.0001). Each group was also significantly different from the other groups (log-rank *P* values < 0.0001). Diabetes incidence during the first follow-up year was 0.7, 1.5, 2.9, 9.2, 30.4, and 44.0% for the baseline HbA_1c_ ≤ 5.5, 5.6–5.7, 5.8–5.9, 6.0–6.1, 6.2–6.3, and 6.4% groups, respectively. Incidence over the entire follow-up period was 3.6, 8.9, 13.8, 27.5, 51.6, and 67.8% for the same groups. The increment of incidence was steeper in the two groups with HbA_1c_ ≥ 6.2% (6.2–6.3 and 6.4% groups), especially in the first follow-up year (*p* ≤ 0.0093, S1 Text). Since the rate of revisit in each subsequent year was greater than 99% in every HbA_1c_ group (data not shown), and because rate of loss to follow-up over 5 years did not differ significantly among HbA_1c_ groups (S2 Table), incidence rates would not be biased by participation rate.

A Cox proportional hazards model was used to evaluate risk of developing diabetes during the follow-up period (Table 2). After adjusting for age, sex, BMI, hypertension, family history of diabetes, exercise habit, smoking habit, and heavy alcohol consumption, the hazard ratio increased with increasing HbA_1c_: 2.3 (95%CI 2.0–2.5), 3.4 (95%CI 2.9–3.7), 8.8 (95%CI 8.0–10.1), 26.3 (95%CI 23.3–30.1), and 48.7 (95%CI 40.8–58.1) for the five ordered groups (compared to the reference group), respectively.

**Table 2:** The risk of diabetes incidence based on the baseline HbA1c levels in the Cox proportional-hazard model.

			HbA_1c_ groups				
	≤5.5%	5.6/5.7%	5.8/5.9%	6.0/6.1%	6.2/6.3%	6.4%	P value
n	34616	9388	4664	2338	1257	518	
Incidence of diabetes (%)	1242 (3.6)	839 (8.9)	645 (13.8)	644 (27.5)	648 (51.6)	351 (67.8)	
Incidence rate per 1000 person-years*	8.7	24.2	39.5	83.4	165.2	242.5	
95% CI	7.3–10.3	19.9–28.6	32.3–46.7	68.1–98.8	137.3–193.2	201.4–283.5	
Unadjusted hazard ratio	1.0	2.1	3.2	8.9	26.8	49.3	<0.0001
95% CI	-	1.90–2.30	2.8–3.6	7.9–9.9	23.7–30.2	41.4–58.4	
Multivariable adjusted hazard ratio ^†^ *	1.0	2.3	3.4	8.8	26.3	48.7	<0.0001
95% CI	-	2.0–2.5	2.9–3.7	8.0–10.1	23.3–30.1	40.8–58.1	

*Actual person-years in each HbA1c group were 142,029, 34,614, 16,277, 7,718, 3,923, and 1,447.

^†^Adjusted for age (≤60,60–69, ≥70), sex, body mass index (BMI), hypertension, family history of diabetes, exercise habit, smoking habit, heavy alcohol consumption. The *p* values of interaction terms were 0.07, 0.37, 0.49, 0.62 and 0.14 for (HbA1c x age), (HbA_1c_ × sex), (HbA_1c_ × BMI), (HbA_1c_ × hypertension), and (HbA_1c_ × smoking habit), respectively.

95% CI: 95% confidence interval; HbA_1c_: hemoglobin A1c

In order to evaluate the multiplicative effect of other factors on HbA_1c_, interaction terms between baseline HbA_1c_ and other risk factors were included in the Cox model (Table 2). Interaction between HbA_1c_ and age was marginally significant (*p* = 0.07). We also calculated the risk after stratification by each risk factor, age (≤60, 61–69, ≥70), sex (male or female), BMI (<24, ≥24 kg/m^2^), hypertension (presence or absence), or smoking habit (presence or absence), one by one in the Cox model (Table 3). Compared with the reference group (HbA_1c_ ≤ 5.5%), the risk for incident diabetes increased steadily with increasing baseline HbA_1c_ but the risk did not differ between stratified subgroups.

**Table 3:** The risk of diabetes incidence based on the baseline HbA1c levels after the stratification of major risk factor in the Cox proportional-hazard model.

HbA_1c_ groups						
	≤ 5.5%	5.6/5.7%	5.8/5.9%	6.0/6.1%	6.2/6.3%	6.4%
**Age (years)**						
≤ 60	1	1.9 (1.7–2.2)	3.0 (2.6–3.3)	8.1 (7.4–8.7)	23.1 (20.0–26.2)	42.5 (37.2–47.8)
61–69	1	1.9 (1.7–2.1)	3.2 (2.8–3.6)	8.9 (7.9–9.8)	27.4 (24.1–30.9)	50.1 (43.6–56.7)
≥ 70	1	2.2 (2.0–2.4)	3.3 (3.0–3.7)	9.5 (8.5–10.6)	29.3 (25.9–32.8)	53.9 (47.0–60.8)
**Sex**						
Female	1	2.4 (2.0–2.7)	3.8 (3.2–4.4)	9.8 (8.6–11.2)	29.8 (26.3–33.5)	58.9 (50.4–67.5)
Male	1	2.6 (2.3–2.9)	4.3 (3.7–4.9)	9.6 (8.3–11.0)	28.6 (24.8–32.1)	50.6 (42.1–59.1)
**BMI (kg/m** ^**2**^ **)**						
< 24.0	1	1.8 (1.6–2.0)	2.8 (2.4–3.2)	8.2 (7.5–9.0)	23.8 (20.8–26.8)	38.5 (34.4–42.6)
≥ 24.0	1	2.0 (1.8–2.2)	3.0 (2.6–3.4)	8.1 (7.4–8.9)	24.4 (21.2–27.6)	39.9 (35.0–44.8)
**Hypertension**						
No	1	1.9 (1.7–2.1)	2.8 (2.5–3.2)	7.6 (6.9–8.3)	19.1 (17.0–21.3)	34.5 (30.7–38.3)
Yes	1	2.3 (2.1–2.5)	3.3 (3.0–3.7)	8.5 (7.8–9.2)	22.3 (20.1–24.6)	37.6 (33.5–41.7)
**Smoking habit**						
No	1	1.6 (1.5–1.8)	2.8 (2.4–3.2)	8.0 (7.3–8.7)	18.1 (16.1–20.1)	32.7 (28.5–36.9)
Yes	1	1.9 (1.7–2.1)	3.4 (2.9–3.8)	9.5 (8.4–10.5)	21.7 (19.6–23.8)	39.8 (35.0–44.6)

Values are expressed as adjusted hazard ratios (95% confidence intervals) calculated by Cox proportional-hazard models.

The analyses were adjusted by age, sex, BMI, hypertension, family history of diabetes, exercise habit, smoking habit, and heavy alcohol consumption except each factor of stratification.

## Discussion

In this large-scale cohort study, we found that baseline HbA_1c_ levels were highly informative in predicting subsequent development of type 2 diabetes. Baseline HbA_1c_ level was an independent risk factor for developing diabetes, and the risk increased with increasing baseline HbA_1c_ levels. The hazard ratios exceeded 26 and 48 in the HbA_1c_ 6.2–6.3% and 6.4% groups, respectively, compared with the normal glucose group (HbA_1c_ ≤5.5%). To our knowledge, this is the first study to analyze incidence of diabetes for baseline HbA_1c_ intervals as narrow as 0.2%.

In Table 4 and Table 5, we compared our results with those of five previous studies that demonstrated the value of HbA_1c_ for predicting onset of diabetes [8,10–12,14]. Among these, our study had the largest sample size (n = 52,781) for both genders. Two other large studies with more than 10,000 subjects had a predominance of either males or females [12,14]. Although all studies consistently showed that risk increases with increasing baseline HbA_1c_ level, the magnitude of incidence in the first year differed from study to study. Individuals with a baseline HbA_1c_ of 6.0–6.4% had an incident rate of diabetes of 12–30% in the first year and of 43–66% in 5 years [12,14]. Since case detection criteria were similar in the latter three studies, differences in ethnicity, gender, mean age, and risk factors could influence the magnitude of the incidence rates.

**Table 4:** Characteristics of the studies which evaluated the incidence of type 2 diabetes using baseline HbA_1c_ levels.

Study	Year of study initiation	Sample size (men %)	Source of subject	Race	Mean age (range)	Follow-up years	Definition of incident diabetes
Edelman et al. [8]	Oct. 1996	1197 (94)	Veterans Affairs Medical Center, USA, Prospective Cohort	White 69%, Black 29%, Others 2%	55 (45–60)	3	HbA1c ≥7.0%, FPG ≥126 mg/dl/l, or self-report
Cheng et al. [14]	Jan. 2000	12375 (95.4)	Veterans Affairs Medical Center, USA, Historical Cohort	White 67.5%, Black 5.8%, Others 1.4%, Unknown 25.4%	65.9* (18.5–101.5)	8	ADA criteria for the diagnosis of diabetes, or treatment for diabetes
Pradhan et al. [12]	Nov. 1992	26563 (0)	Women’s Health Study, USA, Prospective Cohort †	Non-hispanic white 94.8%	54.6 (45–)	10.8	FPG ≥126 mg/dl/l or treatment for diabetes
Shimazaki et al. [11]	Apr. 1996	2820 (49)	Medical care in university hospital, JPN, Historical Cohort	Not-listed ‡	Not-listed§ (15–)	3	Initiation of the anti-diabetic drug
Kogawa-S et al. [10]	Jan. 2008	6804 (100)	Routine health check up, JPN, Prospective Cohort	Japanese 100%	47.7 (40–55)	4	FPG ≥126 mg/dl or treatment for diabetes
Our study	Jan. 2008	52781 (41.4)	Routine health check up, JPN, Historical Cohort	Japanese 98.7%, Others 1.3%	67* (20–89)	5	HbA_1c_ ≥6.5%, FPG ≥126 mg/dl, CPG ≥200 mg/dl, or treatment for diabetes

* Median age

^†^ This study advocated that it was a prospective cohort, but it was a part of a randomized clinical trial.

^‡^ Race was not listed, but almost 100% of participants might be Japanese.

^§^ Mean age was not listed, but 15–39yr., 40–59yr., and ≥59yr. were 24, 32, 44%, respectively.

FPG: fasting plasma glucose; CPG: casual plasma glucose

**Table 5:** Characteristics of the studies which evaluated the incidence of type 2 diabetes using baseline HbA_1c_ levels.

study							
Edelman et al.	**baseline HbA**_**1c**_ **(%)**	**5.1–5.5**		**5.6–6.0**		**6.1–6.5**	
	Incidence at 1 year (%)	0.90		2.53		6.41	
	Incidence at 3 years (%)	2.70		7.59		19.23	
Cheng et al.	**baseline HbA**_**1c**_ **(%)**	**5.0–5.4**		**5.5–5.9**		**6.0–6.4**	
	Incidence at 1 year (%)	4		12		30	
	Incidence at 5 years (%)	13		34		66	
Pradhan et al.	**baseline HbA**_**1c**_ **(%)**	**5.0–5.4**		**5.5–5.9**		**6.0–6.4**	
	Incidence at 1 year (%)	0.1		3		12	
	Incidence at 5 years (%)	2		15		43	
Shimazaki et al.	**baseline HbA**_**1c**_ **(%)**	**≤ 5.5**		**5.6–6.4**			**≥ 6.5**
	Incidence at 1 year (%)	0.15		2.30			16.18
	Incidence at 3 years (%)	0.75		6.90			48.54
Kogawa-Sato et al.	**baseline HbA**_**1c**_ **(%)**	**≤ 5.3**		**5.4–5.8**		**5.9–6.3**	**6.4–6.8**
	Incidence at 1 year (%)	0.8		2.2		6.9	14.0
	Incidence at 4 years (%)	3.0		6.5		20.6	41.9
Our study	**baseline HbA**_**1c**_ **(%)**	**≤ 5.5**	**5.6–5.7**	**5.8–5.9**	**6.0–6.1**	**6.2–6.3**	**6.4**
	Incidence at 1 year (%)	0.7	1.5	2.9	9.2	30.4	44.0
	Incidence at 5 years (%)	3.6	8.9	13.8	27.5	51.6	67.8
Our study *	**baseline HbA1c (%)**	**≤ 5.5**		**5.6–5.9**		**6.0–6.4**	
	Incidence at 1 year (%)	0.6		1.9		20.1	
	Incidence at 5 years (%)	3.6		10.6		46.4	

* We fractionated baseline HbA_1c_ levels into ≤5.5%, 5.6–5.9% and 6.0–6.4%, and evaluated the incidence of diabetes in the same study.

FPG: fasting plasma glucose; CPG: casual plasma glucose

Previous studies evaluated the incidence of diabetes based on intervals of HbA_1c_ of width 0.5% or greater [8,10–12,14]. The cumulative incidence of diabetes from pre-diabetic stage differed greatly from 2.3% to 18.1% per year [8–14]. Our study clearly demonstrated that the incidence of diabetes elevates significantly with increasing baseline HbA_1c_ even between intervals as narrow as 0.2%. Even if an individual is diagnosed as pre-diabetes based on the levels of HbA_1c_ ranging from 5.6 to 6.4%, the subsequent incidence of diabetes differs widely depending on the level of HbA_1c_ within that range. Therefore, it is important and beneficial for clinicians to fractionate baseline HbA_1c_ into narrower groups in the 5.6–6.4% range so as to estimate more accurate incidence of diabetes in each group.

As for other risk factors, we obtained results similar to those reported by others. Conventional risk factors such as male sex [14,19], increasing BMI [20,21], hypertension [14,19,20], family history of diabetes [20,22], non-exercise habit [23–25], smoking habit [20,26–28], and heavy alcohol consumption [20,22,29] were confirmed to be significant risk factors for incidence of diabetes.

It is obvious that participants with higher levels of HbA_1c_ will sooner pass the threshold of diabetes (HbA_1c_ ≥ 6.5%, fasting plasma glucose ≥126 mg/dl, or casual glucose ≥200 mg/dl). In this context, individuals with HbA_1c_ ≥ 6.2% showed a higher incidence of diabetes in the first follow-up year compared with those in later follow-up periods (P ≤ 0.0093). Our results therefore suggest that lifestyle changes should be implemented immediately after observing a result of HbA_1c_ ≥ 6.2%.

We adopted the Japanese guideline that HbA_1c_ levels between 5.6 and 6.4% correspond to a pre-diabetic stage [17,30]. A fasting plasma glucose of 110 mg/dl and a 2-h postprandial plasma glucose of 140 mg/dl correspond to an HbA_1c_ of 5.6% and a fasting plasma glucose of 126 mg/dl and a 2-h postprandial plasma glucose of 200 mg/dl correspond to an HbA_1c_ of 6.5% [30,31]. We also defined diabetes onset as a single result of an HbA_1c_ ≥ 6.5%, fasting plasma glucose ≥126 mg/dl, casual plasma glucose ≥200 mg/dl, or prescription of any medication for treatment of diabetes. The ADA and JDS guidelines recommend that a repeated examination [18,31] and an HbA_1c_ test plus plasma glucose check (fasting, 2-h of oral glucose tolerance test and/or casual) [17] to be conducted for diagnosis of diabetes. However, the JDS has also expressed that for the purpose of estimating frequency of diabetes in an epidemiological survey, “diabetes mellitus” can be substituted for the determination of “diabetic type” from a single examination [17]. The possibility that an individual with either HbA_1c_ ≥ 6.5%, fasting plasma glucose ≥126 mg/dl, or casual plasma glucose ≥200 mg/dl is actually non-diabetic, if any, is slight. We propose that treating them as diabetic patients after a single result of examination is beneficial to them and to society.

There are several limitations to this study. Firstly, the participants were generally old (median age 67 years old) and were Japanese. Thus, generalization to a younger population or to other ethnic groups should be made with caution. Secondly, there was a considerable proportion of individuals who did not revisit (n = 5,489 or 9.0%). However, there were no significant differences in baseline HbA_1c_ levels between the follow-up and the non-revisit groups (S1 Table). Therefore, we believe that non-revisit was random and the current results should not be biased. Thirdly, there were 935 participants who were enrolled into the study but had missing values for participants’ records, or who were lost to follow-up before diagnosis of diabetes after at least one revisit during the follow-up period. They were assigned to the non-diabetes group and included in the analysis. Demographic and clinical characteristics of these individuals were quite similar to the remaining cohort members (S2 Table). Although their absence from the cohort can introduce bias in the ascertainment of both exposure and outcome, we believe that both the small number of these individuals and their demographic characteristics make it unlikely that this substantially affected the reported incidence of diabetes. Lastly, sample-size in the higher HbA_1c_ categories was not adequately large.

In conclusion, the present study indicates that the use of 0.2% intervals of baseline HbA_1c_ increases the accuracy of estimates of incidence of type 2 diabetes. Risk of developing diabetes increased with increasing HbA_1c_ level, especially with HbA_1c_ ≥ 6.2% in the first follow-up year. Compared with previous studies, this study provides more accurate estimates of the changes in the incidence of diabetes as baseline HbA_1c_ levels change. We hope that clinicians can obtain precise risk of diabetes development for pre-diabetic patients by referring to the results of this study, and that they can use these results to lower the risk of diabetes.

## Supporting Information

S1 Table The distribution of baseline HbA_1c_ levels in the follow-up and non-revisit individuals. (DOCX)

S2 Table The distribution of baseline HbA_1c_ levels in the follow-up and lost to follow-up participants (DOCX)

S1 Text Test of the risk difference among first follow-up years. (DOCX)
