# Supplementary material for: Incidence of Type 2 Diabetes in Pre-Diabetic Japanese Individuals Categorized by HbA1c Levels: A Historical Cohort Study
Source: PLoS One. 2015 Apr 8;10(4):e0122698. doi: 10.1371/journal.pone.0122698 (PMC4390315; doi:10.1371/journal.pone.0122698)
Supplement: S1 Table — (DOCX) [file pone.0122698.s001.docx]

**Table S1. Distribution of baseline HbA_1c_ levels in the follow-up and non-revisit individuals**

| **Baseline HbA_1c_** | **≤ 5.5%** | **5.6/5.7%** | **5.8/5.9%** | **6.0/6.1%** | **6.2/6.3%** | **6.4%** | Total |
| --- | --- | --- | --- | --- | --- | --- | --- |
| Follow-up group | 34616 (65.6%) | 9388 (17.8%) | 4664 (8.8%) | 2338 (4.4%) | 1257 (2.4%) | 518 (1.0%) | 52781 |
| Non-revisit group | 3687 (67.2%) | 917 (16.7%) | 446 (8.1%) | 219 (4.0%) | 153 (2.8%) | 67 (1.2%) | 5489 |
| p value | 0.286 | 0.094 | 0.104 | 0.147 | 0.069 | 0.094 | - |

Since there was a considerable proportion of participants who did not revisit (n =5489), we evaluated the distribution of baseline HbA_1c_ levels among them. There were no significant differences in baseline HbA_1c_ levels between the follow-up and the non-revisit groups
