# Supplementary material for: Incidence of Type 2 Diabetes in Pre-Diabetic Japanese Individuals Categorized by HbA1c Levels: A Historical Cohort Study
Source: PLoS One. 2015 Apr 8;10(4):e0122698. doi: 10.1371/journal.pone.0122698 (PMC4390315; doi:10.1371/journal.pone.0122698)
Supplement: S2 Table — (DOCX) [file pone.0122698.s002.docx]

**Table S2. Distribution of baseline HbA_1c_ levels in the follow-up and lost to follow-up participants**

| **Baseline HbA_1c_** | **≤ 5.5%** | **5.6/5.7%** | **5.8/5.9%** | **6.0/6.1%** | **6.2/6.3%** | **6.4%** | Total |
| --- | --- | --- | --- | --- | --- | --- | --- |
| Follow-up group | 34616 (65.6%) | 9388 (17.8%) | 4664 (8.8%) | 2338 (4.4%) | 1257 (2.4%) | 518 (1.0%) | 52781 |
| Lost to follow-up group | 579 (61.9%) | 182 (19.5%) | 95 (10.2%) | 47 (5.0%) | 18 (1.9%) | 14 (1.5%) | 935 |
| p value | 0.282 | 0.270 | 0.199 | 0.402 | 0.374 | 0.119 | - |

935 participants were assigned to the non-diabetes group who had missing values for participants' records or who were lost to follow-up after at least one revisit before diagnosis of diabetes. Demographic and clinical characteristics of these individuals were similar to other cohort members
