# Supplementary material for: Incidence of Type 2 Diabetes in Pre-Diabetic Japanese Individuals Categorized by HbA1c Levels: A Historical Cohort Study
Source: PLoS One. 2015 Apr 8;10(4):e0122698. doi: 10.1371/journal.pone.0122698 (PMC4390315; doi:10.1371/journal.pone.0122698)
Supplement: S1 Text — (DOCX) [file pone.0122698.s003.docx]

**Supplementary text**

**Test of the risk difference among first follow-up years**

We reached the conclusion that incidence of diabetes in HbA_1c_ ≥ 6.2% groups (6.2–6.3% and 6.4%) was significantly higher in the first year of follow-up compared with the other periods (P≤ 0.0093) as follows: First, we divided all participants into 10 intervals in units of 0.1% of HbA_1c_: ≤ 5.5, 5.6, 5.7, 5.8, 5.9, 6.0, 6.1, 6.2, 6.3, and 6.4%. Then, we used the Cochran-Armitage trend test to evaluate whether the incidence of diabetes goes up or down year after year in each HbA_1c_ group. As a result, we found that the incidence of diabetes declined with follow-up year in the HbA_1c_ ≥ 6.2% groups (p<0.05). Next, we compared the incident diabetes of the first year with that of subsequent years using the chi-square test. Because this involves four tests, p values <0.0125 (=0.05/4 by the Bonferroni correction) were considered to indicate statistical significance.

In the HbA_1c_ 6.4% group (n=518), the incidence of diabetes during the first and second years was 43.9% (227/518) and 28.5% (83/291), respectively (p=0.00352). Since annual incidence of diabetes every year after was lower than that of the second year, p values for all subsequent years were lower than 0.00352. In the HbA_1c_ 6.3% group (n=542), the incidence of diabetes in the first year vs. that of the second year was 30.2% (164/542) vs. 18.5% (70/378), respectively (p=0.00174). Since every incidence after the second year was lower than that of the second year, p values were lower than 0.00174 comparing the first and second years. In the HbA_1c_ 6.2% group (n=715), the incidence of diabetes in the first year vs. that of the second year was 8.9% (64/715) vs. 5.1% (33/651), respectively (p=0.00930). Since every incidence after the second year was lower than that of the second year, p values were lower than 0.0093 comparing the first and second years.

Therefore, the highest p value with statistical significance (p <0.0125) was 0.0093, and the individuals with HbA_1c_ ≥ 6.2% had a higher incidence of diabetes in the first follow-up year compared with other follow-up periods (P≤ 0.0093), as described in the main text.
